# Discovering the Lost Reward: Critical Locations for Endocannabinoid Modulation of the Cortico–Striatal Loop That Are Implicated in Major Depression

**DOI:** 10.3390/ijms22041867

**Published:** 2021-02-13

**Authors:** Sari Goldstein Ferber, Aron Weller, Gal Yadid, Alexander Friedman

**Affiliations:** 1 Department of Psychology and the Leslie and Susan Gonda (Goldschmied) Multidisciplinary Brain Research Center, Bar Ilan University, Ramat Gan 5290002, Israel; sari.goldstein@biu.ac.il (S.G.F.); aron.weller@biu.ac.il (A.W.); 2 The Mina and Everard Goodman Faculty of Life Sciences and the Leslie and Susan Gonda (Goldschmied) Multidisciplinary Brain Research Center, Bar Ilan University, Ramat Gan 5290002, Israel; yadidg@mail.biu.ac.il; 3 Department of Biological Sciences, University of Texas at El Paso, El Paso, TX 79968, USA

**Keywords:** endocannabinoid receptors, dopamine, synaptic plasticity, depression, striosomes, ventral tegmental area (VTA)

## Abstract

Depression, the most prevalent psychiatric disorder in the Western world, is characterized by increased negative affect (i.e., depressed mood, cost value increase) and reduced positive affect (i.e., anhedonia, reward value decrease), fatigue, loss of appetite, and reduced psychomotor activity except for cases of agitative depression. Some forms, such as post-partum depression, have a high risk for suicidal attempts. Recent studies in humans and in animal models relate major depression occurrence and reoccurrence to alterations in dopaminergic activity, in addition to other neurotransmitter systems. Imaging studies detected decreased activity in the brain reward circuits in major depression. Therefore, the location of dopamine receptors in these circuits is relevant for understanding major depression. Interestingly, in cortico–striatal–dopaminergic pathways within the reward and cost circuits, the expression of dopamine and its contribution to reward are modulated by endocannabinoid receptors. These receptors are enriched in the striosomal compartment of striatum that selectively projects to dopaminergic neurons of substantia nigra compacta and is vulnerable to stress. This review aims to show the crosstalk between endocannabinoid and dopamine receptors and their vulnerability to stress in the reward circuits, especially in corticostriatal regions. The implications for novel treatments of major depression are discussed.

## 1. Major Depression: Diagnostic Criteria, Prevalence and Etiology

Major Depressive Disorder is one of the most prevalent mental disorders in the Western world and it is the third leading cause of disability in the world. The prevalence rates range, for most countries, between 8% and 12% of the population [1]. According to the World Health Organization, major depression also shows the greatest disability levels and the largest aligned costs for the community and individuals among the mental and behavioral disorders [2,3]. According to the 5th edition of the Diagnostic and Statistical Manual of Mental Disorders (DSM-5) [4], a major depressive episode is defined as a period of 2 weeks or longer during which there is either depressed mood or loss of interest or pleasure (i.e., anhedonia) and four additional symptoms reflecting changes in activity, e.g., psychomotor agitation, retardation or fatigue, change in sleep patterns and suicidality. This disorder is complex, and its etiology, which may include social and psychological factors such as stressful or traumatic events [5,6], inflammation and microbiome [7,8,9], in addition to epigenetic and genetic ones and age-related factors [10,11,12,13], remains unclear to date [14]. Depressive symptoms may be reduced within several weeks after the start of conventional antidepressants, but treatment resistance concerns one-third of the patients who fail to achieve recovery. Hence, the current pharmacological treatment of major depression is accordingly limited. Depression-induced reduction in seeking rewarding experiences and in satisfaction with a rewarding experience in humans as well as in animal models led the scientific community to place the motivational/anhedonic characteristics in the center of the efforts to understand major depression. This involves a focus on the brain reward circuits and underscores the importance of the dopamine (DA) receptors within these circuits [15,16,17,18,19,20,21,22,23,24].

In this review, we suggest understanding major depression as an erroneous computation of cost and reward values produced by reduced sensitivity to reward. We also aim to show that the endocannabinoid and DA systems interacting within the reward circuit and the critical locations of the DA and CB1 receptors open a window of opportunities for pharmacological treatment with cannabinoids in treatment-resistant cases of major depression.

## 2. Dopamine and Major Depression

### 2.1. Location of DA Activity within Subcortical Circuits

Since the 1960s, depression has been associated with dysfunctions of the serotonin (5-HT)- and norepinephrine-circuits [25,26,27], and the first choice for treatment became SSRIs and SNRIs, followed by the development of advanced pharmacological compounds aimed at the reuptake of serotonin and norepinephrine. However, research using neuroimaging, pharmacological, and electrophysiological methods in humans and animal models has shown distinct dopaminergic abnormalities in major depression [28,29,30,31,32,33]; for a review, see [34]. 

Some studies [35] showed that selective inhibition of DA neurons in the ventral tegmental area (VTA), as well as exposure to chronic mild stress (CMS), results in depression-like phenotypes that are normalized by selective activation of the mesolimbic DA system that mediates reward value [36,37]. Additionally, drugs that enhance DA signaling can have antidepressant effects in individuals experiencing major depression [38], further implicating DA dysfunction in major depression. Moreover, pharmacological and other interventions that block or modulate dopaminergic activity can induce or increase depressive-like symptoms in currently depressed or remitted individuals [39,40] suggesting the need to identify a regulated optimal level of DA to be targeted in these novel treatments.

The corticolimbic DA circuit, with excitatory glutamatergic projections from the medial prefrontal cortex to the basal ganglia, is involved in motivational processes, assignment of reward and cost value [41] and in valence, including subjective value [42] It is susceptible to psychosocial stress [43], which, in turn, is a major trigger for the onset, occurrence and reoccurrence of many psychopathologies including major depression. DA transmission is essential for attribution of incentive salience [44] and prediction of reward occurrence [45]. DA neurons fire in response to reward-associated cues and in cases of positive discrepancy between the reward obtained and the reward expected [45,46,47,48]. Accordingly, depression was associated with reduced reward-related functional connectivity between the medial prefrontal cortex and the striatum [49].

### 2.2. Top-Down Bottom-Up Cortical DA Connectivity with Subcortical Circuits

Emerging evidence suggests that the VTA-medial prefrontal cortex (PFC) pathway serves an opposite function to the VTA-NAc pathway, demonstrating the differential role of distinct VTA DA neuron populations in response to rewarding vs. aversive stimuli in the context of depression [30]. This may be responsible for the negative perceptual bias in major depression, implying a negative experience of positive and neutral events.

Additionally, decreased dopaminergic activity is shown in endophenotypes of major depression, such as anhedonia and reduced motivation, and this downregulation appears to originate via hyperexcitation of the infralimbic PFC–basolateral amygdala–ventral pallidum (VP) pathway and possibly via disrupted synaptic plasticity in the ventral subiculum of the hippocampus–nucleus accumbens pathway [16].

### 2.3. The Excitation-Inhibition Imbalance 

The interfered modulation of excitation–inhibition balance in major depression is further evident in an imbalance in the excitatory function of glutamate vs. the inhibitory function of gamma-aminobutyric acid (GABA) within the striatum. Further inhibitory functions are evident in the dopaminergic positive effect on the organism due to lower metabotropic glutamate receptor activation and expression [50]. Preclinical and clinical evidence implicates glutamatergic system impairments in mood disorders such as major depressive disorder.

Interestingly, over the last 20 years, ketamine, an antagonist of the *N*-methyl-D-aspartate receptor, an ionotropic glutamate receptor, has been shown to have antidepressant properties. Specifically, there is a substantial body of literature comprising anecdotal material and descriptions of uncontrolled and randomized controlled trials addressing the use of sub-anesthetic doses of ketamine for the off-label treatment of major depressive episodes [51,52,53]. Furthermore, the functional changes in glutamatergic neurotransmission have been associated with neuronal morphological remodeling, dendritic retraction, and synaptic reorganization, particularly within cortical areas [54]. It is suggested that in major depression the stimulation which inhibits GABA release upregulates the glutamatergic activity and secondarily the dopaminergic pathways [55]. This points to the importance of the excitation–inhibition balance in DA circulation for better understanding of major depression and assigning appropriate pharmacological treatment to this disorder.

In addition, there appears to be an optimal level of DA receptor firing that may be beneficial for the depressive behavioral and emotional profile, as both attenuation and enhanced dopaminergic neural activity have been found to be beneficial for psychiatric disorders. Recent reports suggest that regulation rather than diminution or increases in dopaminergic level is required for the treatment of major depression [56]. This suggests a view of major depression as a cortico–striatal disconnection or spiral error of connectivity between those regions in major depression.

## 3. The Reward Circuits and Major Depression

### 3.1. The Striatum and the Mesolimbic Area

The DSM defines anhedonia as diminished interest or pleasure in response to stimuli that were previously perceived as rewarding before the development of the disorder [4,16]. The dopaminergic neurons of the VTA are connected to mesolimbic pathways often regarded as the “reward pathway”. The center of this network is the striatum. VTA dopaminergic neuronal activity results in higher levels of DA in the striatum guiding motivation and effort, and any abnormalities in dopaminergic signaling could lead to inappropriate and non-gratified reward-seeking behavior [57,58]. This dopaminergic signaling from the VTA is crucial for the functioning of the dorsal and ventral striatum [59], regions associated with motivational experience and motivated behaviors including expectation for rewards [60,61]. Accordingly, research showed that dams from a rat model of depression are less rewarded by pups compared to control dams, and this is associated with less DA spillover and metabolism, as measured by microdialysis [19].

The cortico–striatal circuit in neuroimaging of humans includes the nucleus accumbens, the ventral medial caudate, and the rostroventral putamen [62], and the central regions activating it are the frontostriatal pathways [62,63,64,65,66]. These pathways are activated by dopaminergic projections from midbrain nuclei (e.g., the ventral tegmental area) to subcortical regions central to the evaluation of the reward (e.g., the ventral striatum, including the nucleus accumbens), and finally leading to cortical decision-making and the modulation of emotionality (e.g., the orbitofrontal cortex, medial prefrontal cortex, anterior cingulate cortex). PET and fMRI studies show that primary (e.g., sensory) and secondary rewards (e.g., monetary rewards, reward receipt, reward properties guessing) increase striatal activity [62,67,68,69,70]. Down-regulation or blocking reward system leads to decreased motivation as well as decreased goal-directed cognitions and behaviors. Behaviorally, this is demonstrated by increased withdrawal and negative emotionality (e.g., depressive states and anhedonia) [71]. 

In recent years, down-regulation of the reward system has been related to major depression symptoms, especially reduced motivation and pro-hedonic behaviors, in humans and rats [72,73]. Alterations in the reward circuits may persist after remission in major depression, pointing to the need to pharmacologically address the neural connectivity in the reward circuits to better treat this disorder [74], especially through receptors which crosstalk with the dopaminergic pathways [71]. Interestingly, a rat model of depression exhibits sub-sensitivity in cocaine-seeking behavior, whereas antidepressant treatment raised their cocaine-induced DA release to the level of controls, resulting in increased cocaine-seeking behavior [75]. Further research is needed to investigate the shift from ventral and medial interneurons to the lateral compartment of the striatum including studies of the striatum gradients [76,77,78,79,80,81,82].

### 3.2. The Striosomal Compartment of Striatum

**Figure 1:**
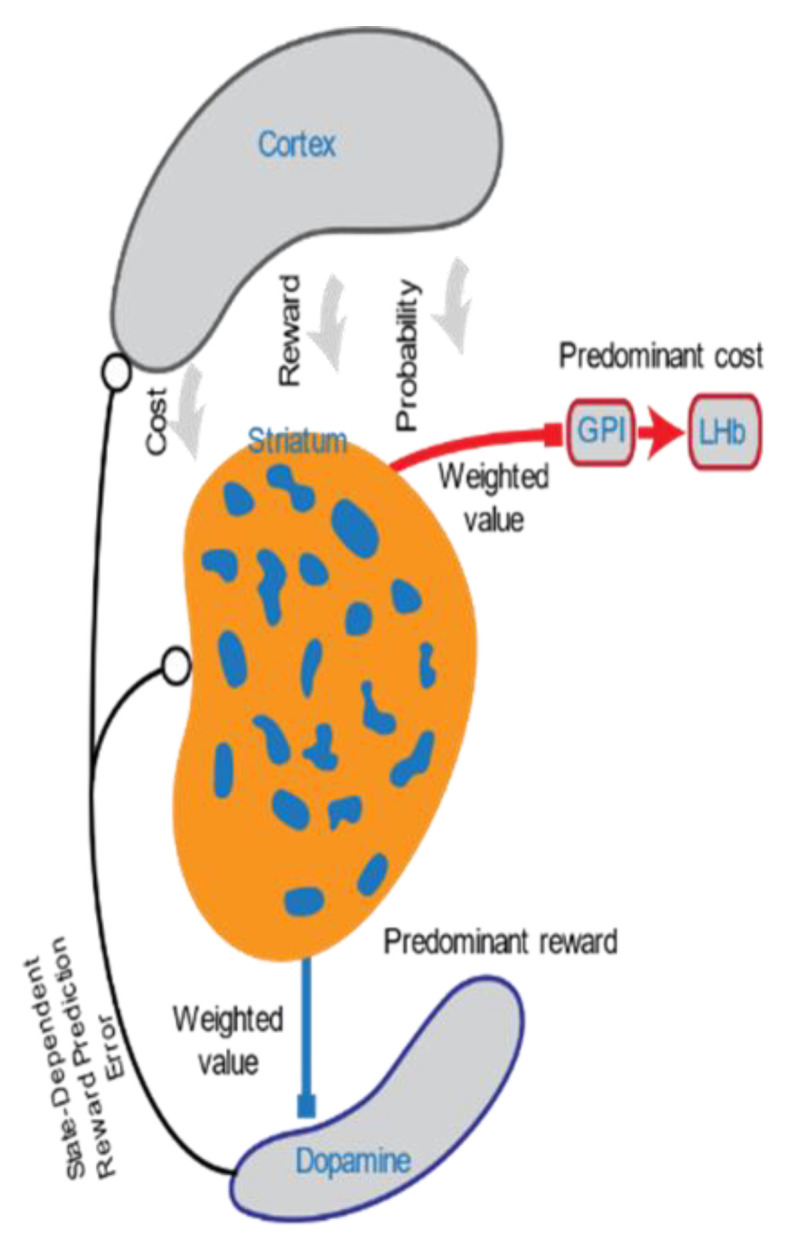
Dorsomedial striatum (DMS) striosomes lie at the center of the Cortico-Striosomal, Lateral Habenula, Dopaminergic Evaluation Circuit. Striosomes encode the subjective value of reward, cost, and uncertainty, and integrate these signals. DMS striosomes selectively project to the SNC (predominant source of reward) and Lateral Habenula.

The striatum has multiple organizing dimensions, including physical three-dimensional regions along ventral–dorsal, medial–lateral and anterior–posterior axes [82,83,84], and its interneurons [85] as well as the projection neurons that give rise to the direct and indirect basal ganglia output pathways [86,87,88,89,90]. A third organizational dimension is represented by the striosomal compartment and the surrounding matrix compartment (Figure 1) [91]. Striatal organization was discovered in 1978 [91]. One compartment of the striatum was called striosomes-bodes in the striatum and the second compartment was called the matrix. It was demonstrated that striosomes receive a distinct set of inputs and outputs [92,93,94]. Major striosomal inputs are prelimbic and infralimbic districts of the prefrontal cortex as well as a orbitofrontal cortex; major outputs of striosomes are dopaminergic neurons of Substantia nigra pars compacta (SNC) (predominant source of reward) and lateral habenula (predominant source of cost) [95,96,97]. Multiple evidence demonstrates that striosomes have molecular and RNA expression that are different from the matrix [98,99].

Of particular behavioral relevance and importance for depression, striosomes receive selective inputs from cortical and subcortical regions related to the limbic system [92,99], and send selective outputs to the lateral habenula (LHb) [96,100,101] and dopaminergic neurons of SNC [95,99,102,103,104], which then ultimately feedback to striatum [105,106,107], including striosomes. This input and output connectivity converts striosomes to a crossroad between limbic districts of cortex and the dopaminergic system. Striosomes, by virtue of their input to LHb and dopaminergic neurons of SNC, could influence the state-dependent modulation of dopaminergic neurons that may be essential for depression.

Recent cell-specific gene expression profiling has demonstrated a striosome-predominant pattern of presynaptic Cannabinoid Receptor 1 (CB1R) expression. The CB1R is abundantly expressed in striatal striosomes and striosome-dendron bouquets of the substantia nigra. Dense CB1R-expressing striosomal fibers extend the substantia nigra pars reticulata, and colocalize with bundles of ventrally extending dendrites of DA-containing SNC neurons [95]. This again shows the relevance of the striosomal circuit for depression.

Aberrant striosomal activity is related to shifts in the dynamic balance of excitation and inhibition in a prefronto-striosomal circuit, resulting in excitation of striosomes in the dorsomedial striatum. The affected circuit elements include neurons of the medial prefrontal cortex and their putative targets in the dorsomedial striatum, including both putative striosomal projection neurons and fast-spiking interneurons. Further radically altered dynamics are apparent in the activity of the fast-spiking interneurons (FSIs), in animals engaged in cost–benefit choices. Importantly, brains of suicide victims, an event often highly associated with major depression, exhibit preferentially increased striosomal expression of prodynorphin, relative to control individuals [108].

## 4. The Endocannabinoid System and Major Depression

The endocannabinoid system (ECS) includes: 1. Endocannabinoids (endogenous ligands), currently characterized: 2-arachidonoylglycerol (2-AG), anandamide (*N*-arachidonoylethanolamine (AEA)), virodhamine (O-arachidonoyl ethanolamine), and *N*-arachidonoyl-dopamine (NADA); 2. Particular enzymes that either degrade or synthesize them; 3. Cannabinoid receptors: the two main ones are CB1R and CB2R; G-protein coupled metabotropic receptors) [109]. Other cannabinoid receptors include G-protein-coupled receptor 55 (GPR55) and transient receptor potential vanilloid type 1 (TRPV1) [110,111,112,113]. CB1R are found in many brain areas and neuron types. CB2R are expressed mainly in the periphery, and the CB2R in the brain [114,115] are not on neurons but rather on microglia [116]. The endocannabinoids exhibit differential affinity in binding CB1R and CB2R as well as to GPR55, GPR18 and GPR119, TRPV1, peroxisome proliferator-activated receptors (PPARs) and glycine receptors [117].

CB1Rs were found in the striatum among other areas. These receptors are also expressed in brain locations of efferent dopaminergic terminals [118]. They were reported to affect excitatory and inhibitory glutamate and GABA synaptic activity, respectively, through cortical afferents and dopaminergic activity. In the dorsal striatum, the ECS exerts long-term presynaptic regulation. The complex role of the ECS in the striatal area is evident in the activation of metabotropic glutamate receptors that can stimulate 2-AG production through activation of phospholipase C [119]. CB1R have been implicated in mediating stress responses in numerous studies, while CB2R are not mentioned frequently in this context [120]. Nevertheless, CB2R have recently been implicated in stress, anxiety and depression [121,122] and their role has yet to be fully characterized.

**Figure 2:**
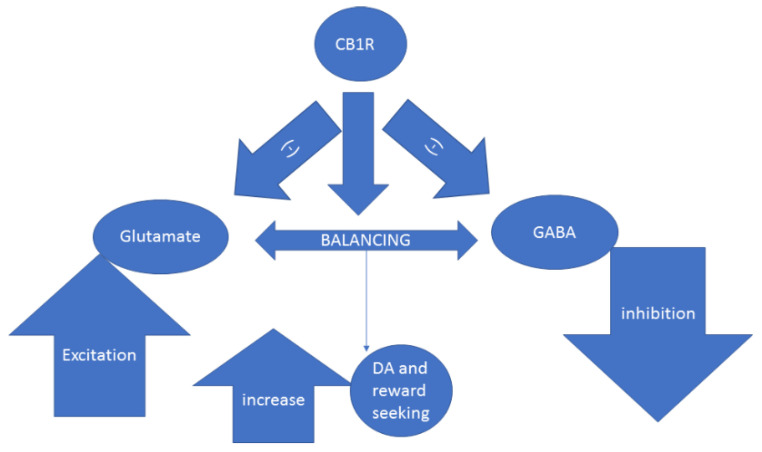
Activation of CB1R in the striatum facilitates glutamatergic and GABAergic balance through inhibition of overshooting which, in turn, increases DA neural activity and reward seeking behavior, pointing to its critical location.

Chronic blocking of CB1R in animals produces anhedonia-like reactions [123]. Animal models also show that compromised CB1R signaling is associated with reduction in sensitivity to reward (sucrose) [124]. The exogenous stimulation of CB1R by exogenous cannabinoids elevated DA release through 2-AG- signaling and CB1R binding [125]. By producing a balanced inhibition on both GABAergic and glutamatergic synapses, the endocannabinoid signaling is able to enhance DA function with the resulting increase in motivation and reward-seeking behavior, as shown in Figure 2 [126]. Due to the potential of the endocannabinoid receptors to increase sensitivity to a reward in the behavioral and neural levels, their location within the reward circuits is crucial for cases of major depression lacking this sensitivity and pro-hedonic behavior related to it [127].

Recently, we discussed the epigenetic fragility of the ECS [128] and the bidirectional function of the system under stress [129]. Moreover, histone deacetylase (HDAC) inhibitors were found to interact with the ECS on the epigenetic level in preclinical studies of depression and chronic stress effects [130,131], suggesting that the crosstalk between ECS and DA receptors may be located at the epigenetic level. The interference of this healthy crosstalk on the epigenetic level may involve further risks for the development of depression, especially under stressful conditions. This has further implications for the development of pharmacogenomics to treat major depression in order to target the availability of ECS-DA receptors beyond monoamine transporters [128].

## 5. The Crosstalk between Dopamine, Striosomes and Endocannabinoid Receptors

CB1R mRNA signal in the ventral and dorsal striatum responds to DA inputs in medium spiny neurons (MSNs) of the striatum [132]. CB1R mRNAs are expressed in both DA 1 receptor (D1R) and D2R-containing cells [133]. Moreover, CB1R were found at the same locations with D2R in pre- and postsynaptic medium spiny neurons of the striatum [134]. It has been shown that endogenous cannabinoids and D2R work in concert and facilitate each other’s neural activity in the brain. Specifically, D2R activity caused the elicitation of anandamide production, which, in turn, was followed by CB1Rs activity in striatum [135].

Accordingly, CB1Rs located on both the GABAergic medium spiny neurons (MSNs) and corticostriatal projections [136,137] are part of the striatal control [138,139], modulating arousal states, as shown in MSNs in animal models of excitotoxicity [140] and Huntington’s disease [141,142,143]. In addition, it has been found that CB1R located in corticostriatal sites differentially protected D1R-MSNs but not D2R-MSNs from cortex-elicited damage by inhibiting glutamatergic activity. Thus, CB1R in corticostriatal projections controls D1R-MSNs of the mouse dorsal striatum by altering glutamatergic output [132]. In summary, the mutual influences between glutamatergic and dopaminergic signaling are considered an important mechanism in major depression [58,144]. Thus, endocannabinoids are an important component that may lead the scientific community towards a better understanding of the potent effects by- and on-dopaminergic activity in the striatum.

## 6. The CB1 Crosstalk with the Striosomal Activity on the Molecular Level

It has been demonstrated that striosomes can be on–off gates on cortico-striosomal circuits [41,43]. An essential level of selectivity is a molecular level underlying this circuit activity, which includes the CB1R. Striosomes, while carrying the supporting value of reward, which is distorted in major depression, produce an enriched CB1R expression via the dendron and through the LHb pathway, suggesting a critical junction for treating major depression. 

Importantly, Friedman and colleagues [42] found a correlation between the activity of the striosomal populations and the valence discriminations achieved by the mice, and these signals and correlations were sensitive to motivational value, as tested by outcome devaluation. Collectively, this and previous studies of striosomal function [41,42] suggest that striosomes could serve as a subjective value filter via integration of cortical task information and motivational state on route to the DA system. 

## 7. Vulnerability of Dopamine and Endocannabinoid Systems within the Reward Circuits: Implications for Major Depression

The treatment of major depression may need to take into account stressful conditions and the reaction to stress within the reward circuits. Inhibition of anti-stress signaling systems within neurocircuits in VP and lateral hypothalamus reduces signaling towards the DA mesolimbic system [126]. The stress effects have a direct influence on DA function, as glucocorticoids modulate the firing of DA neurons [145]. Note that regions involved in the stress reaction are compromised by glucocorticoid mediated atrophy (i.e., hippocampus, PFC) [146] and they are also key regulators of mesolimbic and mesocortical dopaminergic pathways [145] indirectly affecting the reward circuits. The ECS is affected by stress and it only partially recovers with positive inputs [129]. However, recent reports show that the ECS, especially the CB1R, buffers the detrimental impact of stress on reward sensitivity [147,148]. Therefore, the treatment of major depression may need to target stress resilience to reach optimal levels of DA circulation for the subjective experience of reward in the individuals suffering from major depression. More research on the modulation of DA receptors by the endocannabinoid system under stress is warranted. In this regard, measures of PV neurons in the striatum and striatal choline acetyltransferase (ChAT) interneurons, which are sensitive to DA, may emphasize additional critical locations for DA modulation in major depression [107,149,150,151,152,153]. Thus, treatment with cannabidiol (CBD) may need to be based on clinical trials for determining titration rates and to provide the option of monitoring the treatment. 

Depression is a psychiatric disorder that preferentially affects women, and women are at increased risk for developing depression. Since there are sex differences in CB1R availability [154], future research should examine DA-CB1R receptor crosstalk in postpartum depression and sex-dependent DA-CB1R crosstalk in Major Depression, issues still to be clarified.

## 8. Insufficiency of Past and Current Pharmacological Treatments for Major Depression: Implications for Novel Treatment with CBD

The first line of pharmacological treatment for depression, such as serotonin reuptake inhibitors (SSRIs), addresses the monoaminergic systems but is limited by a high resistance rate (e.g., ~40–60% of patients do not reach remission) and delayed onset of therapeutic effects as weeks of treatment are necessary [155,156,157]. This delay particularly underscores the need for fast-acting medication. Anhedonia and reduced motivation, some of the main features of major depression, have been suggested as a cause of treatment resistant conditions [72,127,158,159,160]. This points to the potential therapeutic benefits of treatment with CBD, given its acute positive effects in animal models [161,162].

There is a growing body of knowledge documenting that second-generation antidepressants, such as SSRIs, are not effective in treating positive affect deficits, such as motivation and reward-related cognitive impairment in major depression [163,164]. Moreover, these antidepressants act through 5-HT2C receptors which bind serotonin and inhibit dopaminergic activity. The regulation, rather than inhibition, of dopaminergic pathways may contribute to the suggested low efficacy of SSRIs in treatment-resistant cases due to the necessary firing rate and number of spikes per burst in the reward circuits for the treatment of major depression [165,166,167]. This might underlie the resistance of major depression treated by SSRIs.

Dysregulation in DA expression is also observed in patients treated with typical antipsychotics [168,169], which block D2R [170], leading to DA depletion and a lack of improvement in the ventral striatum [58]. In contrast, the augmentation of treatment with SSRIs by antipsychotic drugs has been shown to increase the expression of DA and the antidepressant effect of SSRIs, thus reflecting the central role of DA expression availability in major depression. This has been shown in humans and animal models and has been approved by the FDA [171]. In addition, ketamine is known to have a strong positive and fast onset efficacy for treating depression; however, it also shows short-term dissociative side effects [16]. Interestingly, ketamine, similarly to atypical antipsychotics, works though modulation of dopaminergic activity in the reward circuits. Due to its problematic side effects, other DA-modulating drugs should be explored.

The literature suggests that CBD acts through DA and serotonin receptors within the mesolimbic areas, especially the VTA and NAc. GABAergic neurons are also implied in this therapeutically effective crosstalk [172]. Specifically, it was found that CBD acts in two phases as a DA partial agonist of D2R [172,173] and attenuating dopaminergic over-production. This suggests the regulatory capacity of CBD. As potentiating anandamide neurotransmission is among CBD’s multiple modes of action [174], its effects on the CB1R can also indirectly improve depressive conditions through regulating excitation–inhibition balance (Figure 1). At a molecular level, regulation of p70S6 kinase (p70S6K) downstream activity by CBD has been found to reverse the effects of amphetamine through dopaminergic pathways [175,176,177]. CBD is an agonist of 5HT1A [178] and DA receptors by the inhibition of cellular reuptake and hydrolysis of the endocannabinoid anandamide [172,179]. The CBD effects on DA-5HT crosstalk in the mesolimbic area may be regulated through the ventral hippocampal area and its connections with other limbic and frontolimbic regions [180]. Thus, this cortico–striatal loop is shown to lead to emotional and cognitive modulation of neuropsychiatric disorders, including major depression. Interestingly, several approved antidepressant treatments upregulate (in most studies) CB1R and DA receptors [117,181,182,183,184]. However, given the high prevalence of treatment resistance, the next generation of pharmacological treatments may need to target the availability of these receptors through pharmacogenomic developments [128].

## 9. For Further Research

New molecular investigations which include RNA sequencing as well as the detection of other critical circuits which function in parallel to the striatum, such as the thalamus, amygdala and the hippocampus, may support the description of a wider scope of relevant locations of CB1R mediating reward in major depression. The involvement of cortical layers in decreasing the sensitivity to reward in major depression and their functionality under CBD treatment may elucidate the critical role of CB1R and pave the way towards future treatment of major depression. Furthermore, the striosomal-CB1R effects, reviewed here, in the rodent’s dorsomedial “associative” striatum, show the striatal region as affected by chronic stress, along with the prefrontal cortex itself [76,185]. While striosomes are not uniform across the striatum in terms of their input–output connections, or in terms of their cell-type constituents, different sub-regions of striatum are selectively affected by chronic stress, thus having complex implications for major depression which warrant further studies [76].

## 10. Conclusions

We showed recent accumulating evidence on the regulation of dopaminergic signaling within the reward circuits by ECS receptors. This has been shown at neural and anatomic levels, as well as at the cellular level. We have also shown that abnormal activity in the reward circuits characterizes major depression. The location of CB1 and DA receptors within the reward circuits, especially the striatal location, is crucial for consideration and development of the next generation of pharmacological treatment of major depression. The crosstalk between CB1, DA and glutamate receptors within the mesolimbic area, especially within the striatum, is accompanied by the stimulation of 5HT in this region, and thus has an upgrading effect for treatment of major depression. Furthermore, we suggested a complex role of CB1R through GABAergic and glutamatergic activity to avoid overshooting or decreasing the dopaminergic activity by stimulation of CB1R. Beyond targeting monoamine transporters, research on pharmacogenomics targeting CB1R availability for treatment of depression and anxiety is warranted [128]. As the ECS is a modulatory and homeostatic network and protects the brain from extreme excitatory or inhibitory conditions, it is suggested that treatment with CBD holds promise for the treatment of major depression. In light of the prevalence of major depression as well as the number of people resistant to current pharmacological treatment, clinical trials of the effects of CBD are warranted.
